# Weathered granites and soils harbour microbes with lanthanide-dependent methylotrophic enzymes

**DOI:** 10.1186/s12915-024-01841-0

**Published:** 2024-02-19

**Authors:** Marcos Y. Voutsinos, Jacob A. West-Roberts, Rohan Sachdeva, John W. Moreau, Jillian F. Banfield

**Affiliations:** 1 School of Geography, Earth and Atmospheric Sciences, The University of Melbourne, Melbourne, VIC Australia; 2 Department of Microbiology, Biomedicine Discovery Institute, Monash University, Melbourne, Australia; 3 Department of Environmental Science, Policy and Management, University of California, Berkeley, CA USA; 4 Department of Earth and Planetary Science, University of California, Berkeley, CA USA; 5 School of Geographical and Earth Sciences, University of Glasgow, Glasgow, UK; 6 Innovative Genomics Institute, University of California Berkeley, Berkeley, CA USA

**Keywords:** Weathered granite, Lanthanides, Rare earth elements, Methanol oxidation, Metallophore, Metagenomics, Mineralogy

## Abstract

**Background:**

Prior to soil formation, phosphate liberated by rock weathering is often sequestered into highly insoluble lanthanide phosphate minerals. Dissolution of these minerals releases phosphate and lanthanides to the biosphere. Currently, the microorganisms involved in phosphate mineral dissolution and the role of lanthanides in microbial metabolism are poorly understood.

**Results:**

Although there have been many studies of soil microbiology, very little research has investigated microbiomes of weathered rock. Here, we sampled weathered granite and associated soil to identify the zones of lanthanide phosphate mineral solubilisation and genomically define the organisms implicated in lanthanide utilisation. We reconstructed 136 genomes from 11 bacterial phyla and found that gene clusters implicated in lanthanide-based metabolism of methanol (primarily *xoxF3* and *xoxF5*) are surprisingly common in microbial communities in moderately weathered granite. Notably, *xoxF3* systems were found in Verrucomicrobia for the first time, and in Acidobacteria, Gemmatimonadetes and Alphaproteobacteria. The *xoxF*-containing gene clusters are shared by diverse Acidobacteria and Gemmatimonadetes, and include conserved hypothetical proteins and transporters not associated with the few well studied *xoxF* systems. Given that siderophore-like molecules that strongly bind lanthanides may be required to solubilise lanthanide phosphates, it is notable that candidate metallophore biosynthesis systems were most prevalent in bacteria in moderately weathered rock, especially in Acidobacteria with lanthanide-based systems.

**Conclusions:**

Phosphate mineral dissolution, putative metallophore production and lanthanide utilisation by enzymes involved in methanol oxidation linked to carbonic acid production co-occur in the zone of moderate granite weathering. In combination, these microbial processes likely accelerate the conversion of granitic rock to soil.

**Supplementary Information:**

The online version contains supplementary material available at 10.1186/s12915-024-01841-0.

## Background

During the weathering of granite, a major component of Earth’s continental crust, some elements are redistributed into new, more stable minerals, a critical process in the formation of soil [1]. Microorganisms are potentially important drivers of mineral weathering, but the mechanisms by which they promote mineral alteration in weathering rock, and the biogeochemical connections between microbial metabolisms and element redistribution are poorly understood.

Many microorganisms harbour mechanisms to derive essential trace metals from the environment. *Methylosinus trichosporium* OB3b uses copper (Cu) as a cofactor and to regulate the expression of particulate methane monooxygenase for the oxidation of methane (CH_4_) to methanol (CH_3_OH) [2]. Copper is released from rocks by natural weathering processes before precipitating as non-bioavailable secondary minerals such as chalcocite (Cu_2_S) (K_spa_ 6 × 10^−16^). Under Cu limiting conditions, *M. trichosporium* OB3b produces chalkophore (methanobactin) [3] to dissolve Cu mineral phases [4]. Chalkophores also have affinities beyond Cu including Cd, Co, Fe, Mn, Ni, Zn and Pb, U [5].

A critically important component of granite weathering is the conversion of mineral-associated phosphate to bioavailable phosphorus. Apatite [Ca_5_(PO_4_)_3_(F,Cl,OH)], the primary phosphate mineral, dissolves early, but much of the phosphorus released is sequestered into secondary phosphate minerals [6]. The major cations in these secondary minerals are often lanthanides that may be released by dissolution of minerals such as allanite (Ca,Ce,La)_3_(Fe^2+^,Fe^3+^)Al_2_O (Si_2_O_4_)(Si_2_O_7_)(OH) and monazite (Ca,Ce,La)PO_4_. The resulting minerals, such as rhabdophane (Ce,La,Nd,PO_4_·H_2_O) and florencite (La,Ce,Nd, Sm,Ba,Ca,Fe,Pb)Al_3_(PO_4_)_2_(OH)_6_, are exceedingly insoluble (< 10^−25^*K*_sp_) [7, 8]. This imposes a nutrient limiting effect on the ecosystem, commonly resulting in infertile soils [9, 10].

Lanthanides were long considered to be of no biological relevance. However, now it is known that some facultative and obligate methylotrophic bacteria oxidise methanol to generate energy using XoxF enzymes [11], a class of lanthanide-dependent methanol dehydrogenases (MDH). These pyrroloquinoline quinone (PQQ)-bound enzymes require lanthanides in their active site to catalyse the reaction of methanol to formaldehyde in the periplasm [12]. Genomic analyses (mostly of isolate genomes) revealed a diversity of putative XoxF enzymes that were suggested to be the dominant form of MDH and evolutionarily older than the more well studied calcium-dependent MDH (MxaF) [11]. To date, there are five known phylogenetically distinct clades of XoxF (XoxF1-5). XoxF5, XoxF4 and XoxF2, XoxF1 are experimentally studied clades, with representative sequences characterised as functional in Alphaproteobacteria *Methylobacterium extorquens* AM1 [13] and in Verrucomicrobia *Methylacidiphilum fumariolicum* solV [14], respectively. Based on metagenomic studies of soil, putative lanthanide-based XoxF3 (hereafter referred to as XoxF3) is present in bacteria from a diverse range of phyla, including Proteobacteria, Acidobacteria, Gemmatimonadetes and Rokubacteria [15, 16]. Sequence alignments demonstrated that these proteins have the conserved amino acid residues required for lanthanide-based functionality [11]. XoxF5 is the largest clade reported to date, and together with XoxF1 and XoxF4, are only reported in Proteobacteria [17].

Many experimental studies [13, 14, 18, 19] and one mini-review [11] indicate that most XoxF-based systems are comprised of the core periplasmic MDH XoxF, and homologues of XoxJ [20] (a periplasmic binding protein of unknown function) and XoxG [21] (a periplasmic membrane bound cytochrome specific to XoxF). XoxF3-based systems have not been experimentally studied, but one mini-review identified that a few *xoxF3* operons include cytochrome genes (*cox*, *ctaG*) [11]. Overall, little is known about the suite of genes involved in *xoxF*-based systems outside of Proteobacteria.

Given that lanthanides in weathered granite are sequestered in highly insoluble minerals, theoretical considerations [19, 22] and recent experimental work [23] suggest that specialised molecules such as metallophores may be required to induce lanthanide release. Metallophores are produced by biosynthetic gene clusters (BGCs) such as nonribosomal peptide synthetases (NRPS) and polyketide synthases (PKS) [24]. A recent preprint reports the first experimentally verified example of lanthanide-associated metallophore production in Alphaproteobacteria *Methyloruburm extorquens* AM1. This organism was shown to upregulate a lanthanide chelation cluster (LCC) when supplied with poorly soluble Nd_2_O_3_ in vitro. LCC encodes a NRPS biosynthetic gene cluster containing a TonB-dependent transporter (TBDT) and synthesises an aerobactin-like siderophore [23]. This LCC is conserved across Methylobacterium species with some components present in other Alphaproteobacteria. Interestingly, soils from which diverse lanthanide-based metabolisms have been inferred contain microbial communities that include organisms with numerous secondary metabolism gene clusters [15, 16, 25, 26]. Thus, it is reasonable to speculate that the capacity to produce secondary metabolites that promote release of lanthanides from phosphate minerals may co-occur with genes for proteins that require lanthanides for functionality.

Despite many studies of microbial processes in soil [27–31], the microbial communities and their genomically encoded functionalities in weathered rock have remained understudied. Nor has any study investigated the lanthanide-solubilising capacity of microorganisms in such environments. The ‘onion skin’ feature of some granites, a concentric progression from weathered material towards freshly exposed rocks, provides an ideal opportunity to study both the mineralogy and microbiology along a weathering profile. Here, we characterised the mineralogical, geochemical and potential microbiological processes occurring in weathered I-type granite and associated soil, with a focus on potential for lanthanide utilisation and secondary metabolism. We report that diverse bacteria can perform lanthanide-based metabolism of methanol in moderately weathered rock, where lanthanide phosphates are solubilised.

## Results

### Sampling across a granite weathering profile

**Fig. 1:**
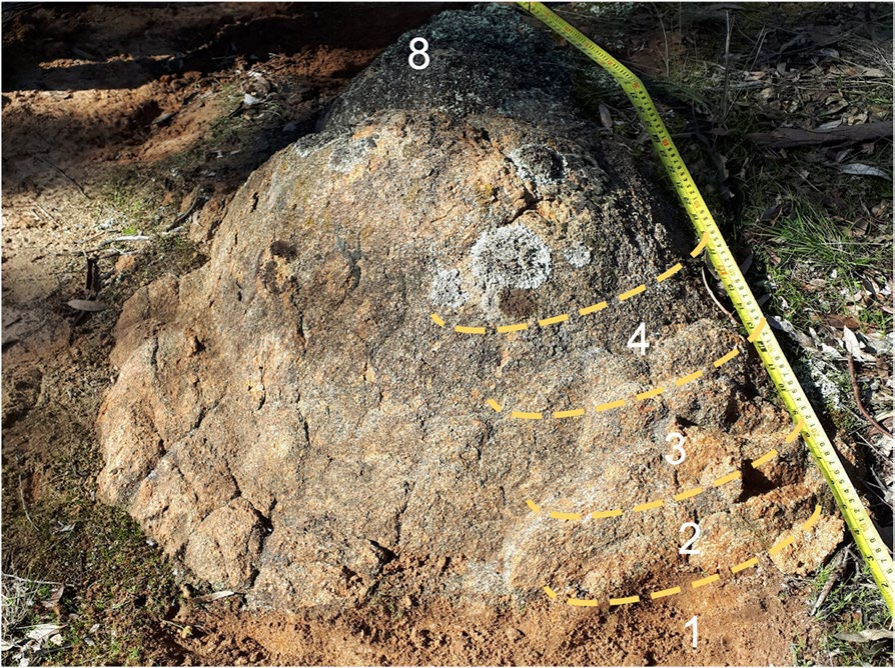
Scale photo of the weathered I-type granite profile. Numbers indicate sampling locations for geochemical samples. Sample 1 is the most weathered sample and sample 8 is the least weathered sample. Photo taken after the first sample was collected

We sampled fresh and weathered I-type Burrumbeep granodiorite and soil from near Rocky Point Bushland Reserve (RPR), Ararat, Victoria, Australia (Fig. 1). The Victorian Geological Survey notes that this Ararat Suite granodiorite contains hornblende, biotite, zircon, apatite, allanite, sphene, calcite, fluorite, chlorite, quartz and plagioclase and K-feldspars [32]. The mineralogy of the samples was confirmed via thin section analysis and scanning electron microscopy-based energy dispersive X-ray analysis (SEM-EDX). The densities of weathered and fresh rocks were measured and compared to provide an indication of the degree of alteration (i.e. mass loss). Samples with densities > 2.5 g/cm^3^ were classified as nearly fresh rock, 2.4 to 2.2 g/cm^3^ as lightly weathered, 2.1 to 1.9 g/cm^3^ as moderately weathered, 1.8 to 1.6 g/cm^3^ as highly weathered and < 1.6 g/cm^3^ as very highly weathered. Highly weathered samples lacking granitic texture were classified as soil. Nine samples were collected for metagenomics analysis, representing moderately weathered rock (1.9 g/cm^3^), highly weathered rock (saprolite; 20 cm below the surface) and soil.

### Microbial community composition as a function of weathering extent

**Fig. 2:**
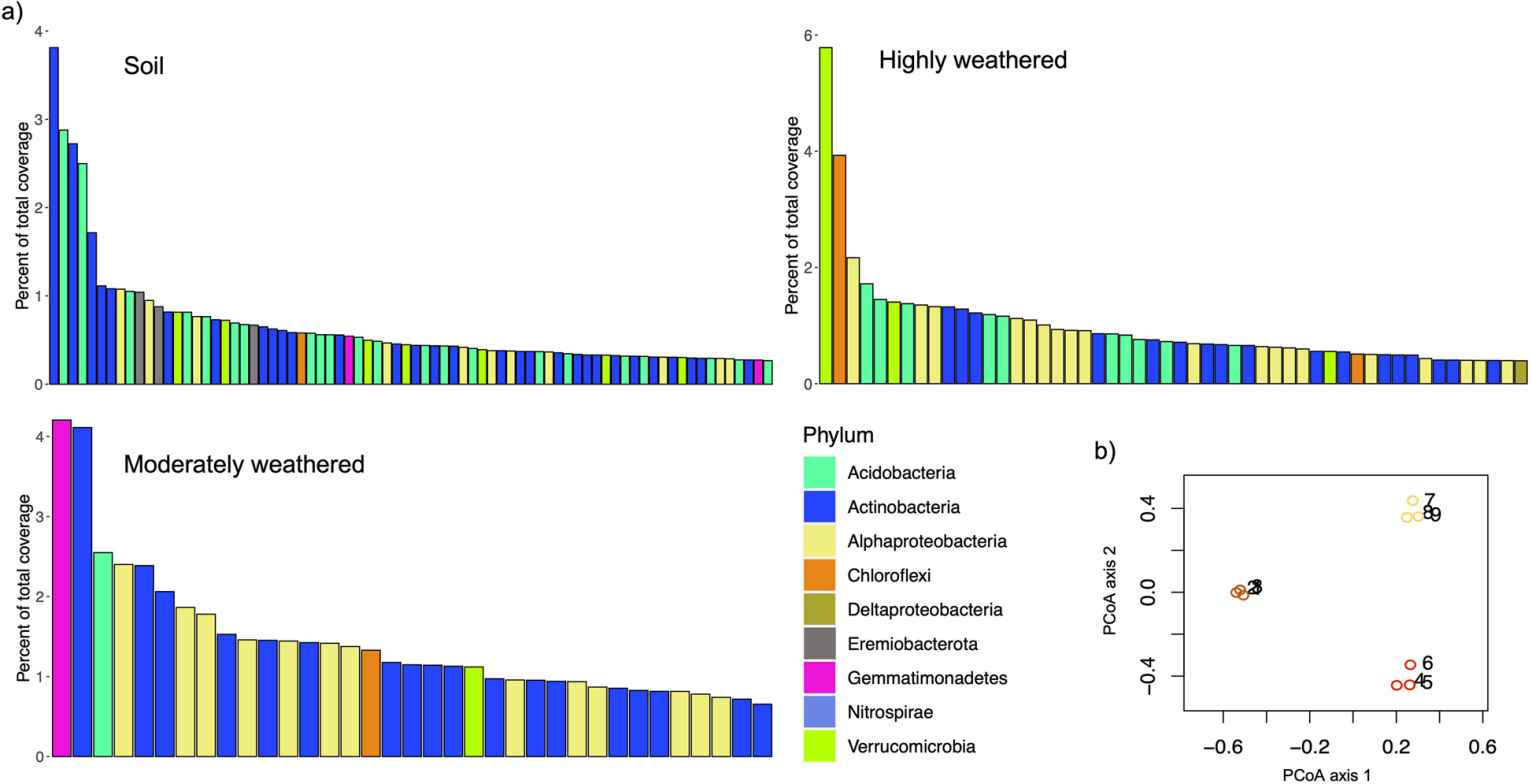
rpS3 species group diversity and abundance throughout a weathered granite profile. **a** Percent of total relative coverage for the top 50% of the ribosomal protein S3 bearing contigs representing species groups. **b** PCoA plot showing beta diversity of the nine communities collected from the soil (yellow), moderately (orange) and highly (red) weathered regions. Refer to Supplementary Table 1 for all species groups recovered and Supplementary Table 2 for their relative abundance

We used ribosomal protein S3 (*rpS3*) gene sequences to assess the microbial diversity of the weathered profiles and used coverage of contigs carrying these genes to quantify relative microbial abundance. The *rpS3* gene is a universal single-copy gene that is a good phylogenetic marker in soils because it assembles well from metagenomic data and is recovered more frequently than 16S rRNA genes [33]. Across our metagenomic assemblies, we identified 3191 rpS3 sequences. All rpS3 sequences longer than 180 aa were grouped into 1231 species groups (Additional file 1: Supplementary Table 1) based on 99% similarity (see Methods). We classified all the rpS3 sequences by constructing a phylogenetic tree containing our sequences and the rpS3 sequences from diverse bacteria [34], and identified organisms from 18 phylum-level lineages (Additional file 2: Supplementary Data 1) and their relative abundance (Additional file 1: Supplementary Table 2). Actinobacteria and Alphaproteobacteria were the most abundant phyla in the moderately weathered region, while Actinobacteria and Acidobacteria were the most abundant in the soil and highly weathered regions. Some bacterial species groups exhibited high relative coverage while their phyla accounted for a small fraction of all the phyla represented in the community. This was most evident in the moderately weathered granite where sometimes only one microbial representative was present from Gemmatimonadetes, Acidobacteria, Chloroflexi and Verrucomicrobia despite exhibiting a high percentage of total coverage (Fig. 2a). This trend was also observed with Verrucomicrobia and Chloroflexi in the highly weathered region and Eremiobacterota, Chloroflexi and Gemmatimonadetes in the soil. Ordination analysis (Fig. 2b) of the coverage data showed that communities sampled from the same weathering zone were more similar to each other than those from other weathered zones.

### Abundance of the xoxF gene relative to degree of weathering

**Fig. 3:**
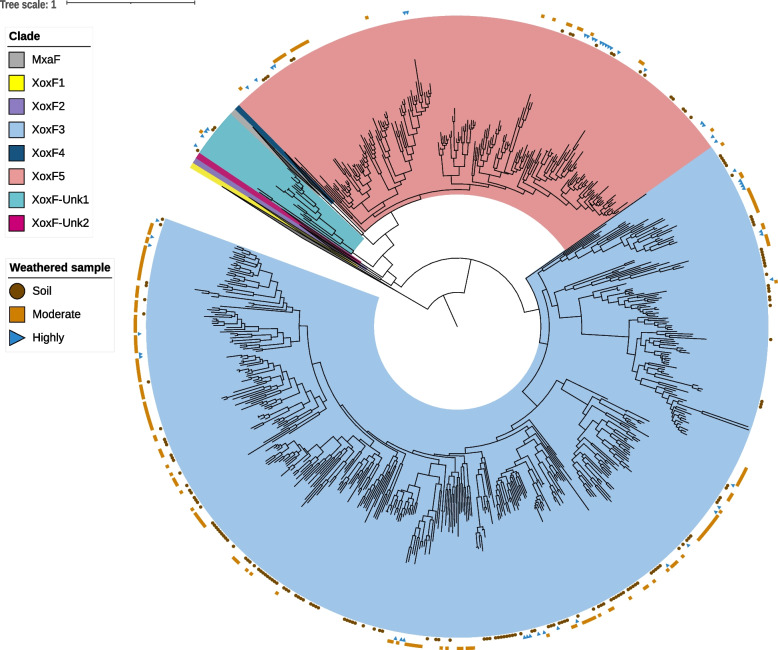
Phylogenetic analysis of the 411 methanol dehydrogenase sequences isolated from the metagenomes of the weathered granite profile. Weathered regions from which sequences were assembled are indicated by an orange square (moderate), brown circle (soil) and blue right triangle (highly). Clades that do not contain sequences from this work are collapsed. Branches are coloured to indicate xoxF clade. Unknown subtypes indicate the references did not have a known clade. PQQ-ADH1 is present as an outgroup

A HMM for PQQ-binding alcohol dehydrogenases was used to detect methanol dehydrogenases encoded on the assembled contigs. Dereplication yielded a set of 411 distinct sequences. All were of the XoxF type and contained the catalytic and cofactor binding residues required for activity, including those for PQQ, as well as conserved aspartate residues for binding lanthanides. No MxaF (Ca-dependent) representatives were identified. XoxF3 was most abundant, with 340 sequences, followed by XoxF5, with 63 sequences and 8 XoxF sequences that could not be assigned to a clade. Overall, XoxF sequences were more commonly assembled in the moderately weathered (187) compared to highly weathered rock (54), and slightly more than in soil (170) (Fig. 3 and Additional file 1: Supplementary Table 3). Thus, our findings extend our knowledge about XoxF in soils by showing that the capacity for lanthanide-dependent methanol oxidation is highly represented in diverse bacteria in weathered rock prior to its conversion to soil.

### Genome reconstruction

We reconstructed 136 non-redundant draft genome sequences (> 70% complete with < 10% contamination) from 11 different bacterial phyla. The most frequently genomically sampled bacterial phyla was Actinobacteria, followed by Acidobacteria and Alphaproteobacteria (Additional file 3: Supplementary Fig. 1). For reasons of their abundance and prior data indicating high lanthanide metabolism and secondary metabolite production capacity [16, 25], we analysed in more detail the phylogeny of the organisms represented by 28 Acidobacteria genomes (Additional file 3: Supplementary Fig. 2). A phylogenetic tree, using 16 ribosomal protein sequences of Acidobacteria genomes from this study and 150 reference genomes from [16], representing many Acidobacteria groups, was built to classify the genomes. The majority were placed within Group 1 Acidobacteriales, Group 3 Solibacteres and Group 4 Blastocatellia.

### XoxF3 diversity and phylogenetic associations

Recent studies have confirmed that an additional aspartate residue absent from the Ca-dependent MxaF enzyme is essential for lanthanide binding and function in XoxF1 [35], XoxF2 [14], XoxF4 and XoxF5 [36]. While our XoxF3 sequences contain the additional aspartate residue no biochemical data are available for this putative lanthanide-based enzyme [37].

**Fig. 4:**
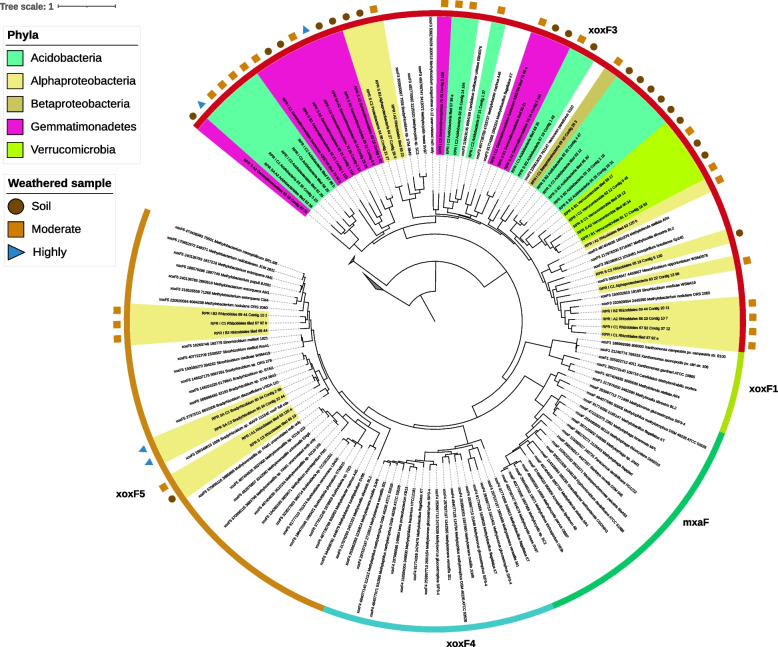
Phylogenetic analysis of the 50 xoxF sequences recovered from the dereplicated genome set and their associated taxonomy and weathered profile. Uncoloured sequence labels are reference XoxF sequences taken from [16]. Sample names are coloured to indicate their representative bacterial phylum

In the set of 136 dereplicated genomes, 43 XoxF3 and 7 XoxF5 sequences were identified. Our genomes did not contain NAD-dependent methanol dehydrogenase. XoxF was found in all regions but was much more common in the genomes of bacteria sampled from moderately compared to highly weathered rock and was also abundant in the genomes of soil bacteria. The XoxF genes were detected in Acidobacteria (gp 1 Acidobacteriia, gp 4 Blastocatellia, gp 2 and gp 3 Solibacteres), Gemmatimonadetes, Verrucomicrobia and Alphaproteobacteria genomes (Fig. 4). XoxF is a periplasmic methanol dehydrogenase, so it is not surprising that XoxF was not detected in any of our MAGs for gram-positive bacteria. All XoxF5 sequences occurred in Alphaproteobacteria genomes. We conclude that XoxF is utilised by bacteria from diverse phyla, is the dominant methanol dehydrogenase and is particularly abundant in moderately weathered rock.

### Finding of XoxF3 systems in Verrucomicrobia

Five high-quality genomes from Verrucomicrobia contained XoxF3 systems (Additional file 3: Supplementary Fig. 3). Two genomes also contain a cysteine-rich copper-binding protein (DUF326) which may chaperone copper to cytochrome c oxidase. Other genes generally co-localising with XoxF3 include a mechanosensitive ion channel, a TBDT (COG 1629), XoxJ (periplasmic binding protein of unknown function) and cytochrome enzymes including XoxG (XoxF specific class I c-type cytochrome) most closely related to Acidobacteria class I c-type cytochrome. To our knowledge, this is the first time a XoxF3 system has been predicted in Verrucomicrobia.

### XoxF3 systems are conserved across Acidobacteria and Gemmatimonadetes

**Fig. 5:**
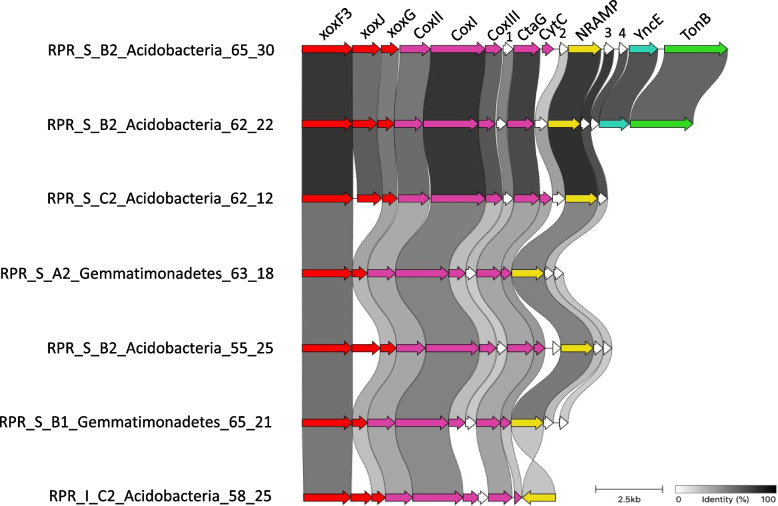
Gene cluster comparison of xoxF3 systems conserved across Acidobacteria and Gemmatimonadetes. Grey links show the percentage of identity between homologous proteins from different genomes. Abbreviations in order of appearance: xox, methanol dehydrogenase; Cox, cytochrome c oxidase; CtaG, cytochrome C oxidase assembly factor; CytC, cytochrome c; NRAMP, natural resistance macrophage protein; YncE, PQQ-dependent catabolism-associated beta-propeller protein; TonB, TonB-dependent receptor. Genes labelled 1, 2, 3 and 4 are hypothetical proteins

Five Gemmatimonadetes genomes contained 13 XoxF3 (Additional file 3: Supplementary Fig. 4) with one genome (RPR_S_B1_Gemmatimonadetes_65_21) containing four XoxF3. Almost all the XoxF3 systems contained XoxG, and four contained XoxJ. All Acidobacteria genomes with XoxF systems contained XoxF3 (12 genomes) co-localised with cytochromes (CoxI, CoxII, CoxIII, CtaG, CytC), XoxG, XoxJ and a small (~ 110 aa) hypothetical protein. Many genomes contained three small (~ 110–130 aa) hypothetical proteins surrounding a natural resistance-associated macrophage protein (NRAMP) domain (Fig. 5). Notably, seven Acidobacteria and two Gemmatimonadetes genomes contained NRAMP in the XoxF3 encoding region. Further, most Acidobacteria and two Verrucomicrobia and Gemmatimonadetes genomes contain XoxF3 systems including a ~ 110–130 aa conserved hypothetical protein between CoxIII and CtaG. The Alphafold2 prediction of the 3D structure of this protein indicates high similarity between these proteins and vesicle-mediated transport proteins (Additional file 3: Supplementary Fig. 5). To our knowledge, neither the NRAMP nor the putative vesicle-mediated transport proteins have previously been associated with XoxF systems.

### Lanmodulin and Pho regulon

Lanmodulin, a periplasmic lanthanide-binding protein that has been experimentally studied in the Alphaproteobacterium *Methylobacterium extorquens* [38], was identified in one Bradyrhizobium (Alphaproteobacteria) genome but was not co-localised with XoxF. This protein contained 3 EF hand domains with the lanthanide-specific proline residues [38]. We also identified another protein containing 3 EF hand domains but the domains contain neither the proline for lanthanide coordination nor the lysine residue for calcium coordination (Additional file 4: Supplementary Data 2). We did not identify other homologues of lanmodulin in other genomes or in the metagenomes in this study.

A complete Pho regulon was identified downstream from XoxF3 (Additional file 3: Supplementary Fig. 6) in the genome of RPR_S_B1_Gemmatimonadetes_65_21. Regulation of the pho regulon is controlled by phoU, phoR and phoB, pstS is a phosphate-binding protein and pstCAB form the transport complex that shuttles phosphate across the inner membrane [39]. This regulon was identified in other Gemmatimonadetes genomes but genome fragmentation prevents clarification of the genomic context relative to the XoxF systems. While close genomic association is not sufficient evidence to link these processes, it is interesting, given that these elements are important nutrients and the strong affinity phosphate has for lanthanides resulting in their precipitation.

### Sources of lanthanides

**Fig. 6:**
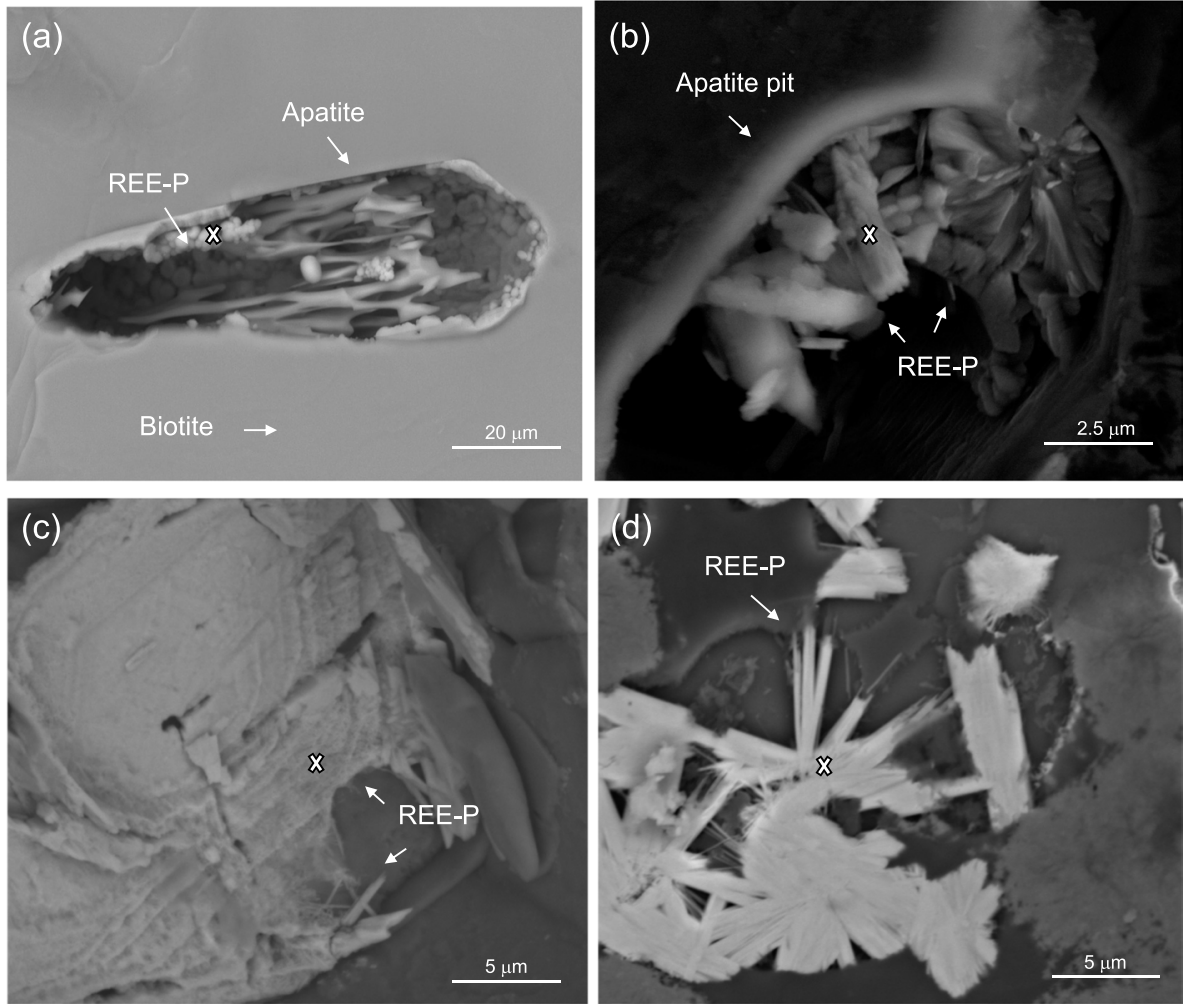
Scanning electron micrographs of secondary lanthanide phosphate minerals **a** replacing apatite (ei19 sp10), **b**, **c** precipitating in relict apatite pits (ei11 sp12 and ei29 sp47) and depositing on the surface of **d** biotite grains (ei33 sp 25). White ‘x’ indicates the site of EDX analysis. For EDX data, refer to Supplementary Table 7. SEM images captured at 10 kV

An analysis of the extent of dissolution and pitting of apatite crystals enclosed within biotite showed that, as expected, increased degree of weathering correlated with increased extent of apatite dissolution (Fig. 6a). Secondary lanthanide phosphate minerals occurred as subhedral to euhedral crystals up to ∼1 μm long and ∼0.2 μm wide in hexagonal pits within biotite that were partly or previously occupied by apatite and as crystal aggregates on the surface of biotite (Fig. 6b–d). Secondary lanthanide phosphates were most abundant in the lightly weathered material, mostly dissolved in the moderately weathered material, and absent in the highly weathered material. This pattern was clarified by whole-rock elemental compositional data (Additional file 3: Supplementary Fig. 7 and Additional file 1: Supplementary Table 4) that showed that total lanthanide concentrations peaked in the lightly weathered (RPR1 - density 2.1 g/cm^3^) granite (427 ppm), where La concentrations were over five times higher than in fresh granite. The high enrichment of lanthanides in lightly weathered rock has been described previously [6, 40] and is attributed to redistribution of lanthanides from more weathered rock into phosphate phases at the weathering front.

Total lanthanide concentrations decrease substantially in the moderately weathered granite (264 ppm). Assuming approximately isovolumetric weathering (consistent with preservation of granitic texture), this supports the interpretation that lanthanides were lost much faster than other constituents in this zone. Some of the released La was likely transported into slightly weathered granite to account for the dramatic enrichment in this zone, yet lanthanides would be available for microbial utilisation. Concentrations were even lower in the highly weathered granite (214 ppm) and soil (80 ppm).

### NRPS/PKS abundance and distribution

Given that siderophore-like molecules are suspected to be important in promoting release of lanthanides, we investigated secondary metabolic capacity in the whole metagenomes (binned and unbinned sequences > 10 kbp). One thousand nine hundred biosynthetic gene clusters were identified (Additional file 3: Supplementary Fig. 8 and Additional file 1: Supplementary Table 5). The amount of DNA sequence that encoded BGCs decreased from 0.53% in moderately weathered rock to 0.34% in the soil. This trend was paralleled by nonribosomal peptide synthetases (NRPS, NRPS-like) and polyketide synthases (PKS types I, II, III and PKS-like). The incidence of predicted metallophores (NRPS/PKS BGCs containing TBDT) was highest in the soil compared to moderately and highly weathered rock. Two other transporters shown to be predictive of metallophore activity [41], FecCD and Peripla_BP_2 did not occur in our samples.

We also predicted biosynthetic gene clusters in the set of 136 dereplicated genomes. We identified 457 biosynthetic gene clusters on contigs ≥ 10 kb and an additional 186 biosynthetic gene clusters on contigs less < 10 kb (Additional file 1: Supplementary Table 6). Of these, 168 were NRPS/PKS gene clusters, and they derived from genomes of bacteria from 10 different phyla (plus 54 smaller and possibly incomplete clusters). Biosynthetic gene clusters on contigs > 10 kb were most abundant in Acidobacteria and Actinobacteria, and most of these were NRPS/PKS systems (Additional file 3: Supplementary Fig. 9). BGCs from genomes were most abundant in the moderately weathered granite while NRPS/PKS abundances were essentially the same in each of the sampled zones (Additional file 1: Supplementary Table 6).

### Metallophore abundance and distribution

**Fig. 7:**
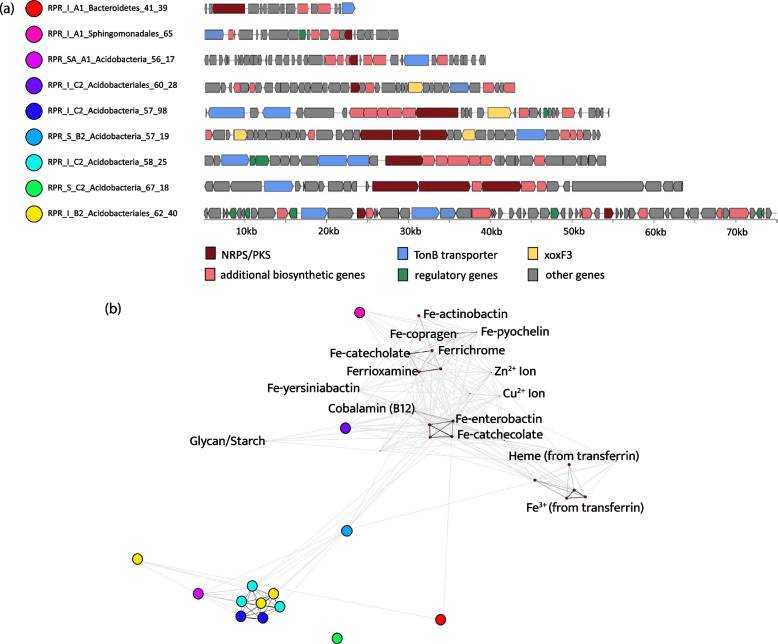
Genomic organisation of the predicted metallophores from methylotrophic MAGs and the relationship of their TBDT with characterised TBDTs. **a** All BGCs with predicted metallophore activity identified in the dereplicated genome set. All BGCs were detected using AntiSMASH 6.0. TonB transporters were identified using pfam_transporter20.hmm and xoxF sequences were detected using a customised HMM for PQQ-binding alcohol dehydrogenases. **b** Sequence-clustering analysis of predicted metallophore TBDTs with TBDTs of known function and/or structure. The TBDTs from each predicted metallophore is represented by a coloured circle

Transporters are required for the import and export of specialised biosynthesised metabolites such as siderophore-like molecules. We identified all classes of transporters across the 136 dereplicated genomes. The metallophore predictive TBDT co-occurred with 8 NRPS/PKS biosynthetic gene clusters (Table 1 and Fig. 7a). Three Acidobacteria (two group 1 Acidobacteriia and one group 2) genomes from the soil and moderately and highly weathered granite contained putative metallophores co-localised in genomes with XoxF3 systems. These XoxF systems occurred inside the antiSMASH predicted NRPS/PKS biosynthetic clusters. A further three Acidobacteria (two group 3 Solibacteres and one group 4 Blastocatellia) genomes from the moderately weathered granite contained XoxF and putative metallophore systems, but these were not co-localised with XoxF. Four genomes contain siderophore-like BGCs, but no XoxF systems were identified. Three of these genomes were Bacteroidetes, Sphingomonadales, Acidobacteria (group 1 Acidobacteriia) from the moderately weathered region and the other was for a Acidobacteria (group 4 Blastocatellia) from the soil. We did not observe any systems similar to the lanthanophore cluster of *Methylorubum extorquens* AM1 [23].

**Table 1:**
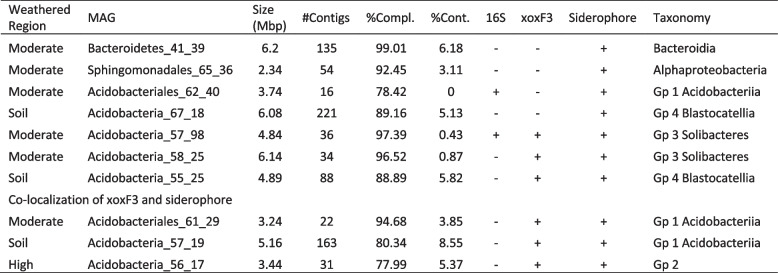
Summary of 10 representative MAGs carrying xoxF3 and/or metallophore

### TonB-dependent transport cluster analysis

TonB-dependent transporters are predictors of siderophore functionality of biosynthetic gene clusters [41]. These transporters are required for transport of specialised metabolites across the outer membrane. TonB receptors for the transport of iron [42], copper [43], zinc [44], polysaccharides [45] and cobalamin (B12) [46] have been characterised. To determine the relationship between TBDTs identified in our putative metallophores, we used CLANS [47] to perform a cluster analysis based on pairwise sequence-similarity against characterised TBDTs (Fig. 7b). The results reveal that no TBDT sequences cluster with characterised TBDTs indicating that they may transport novel metabolites.

### Lanthanide-dependent methylotrophy

**Fig. 8:**
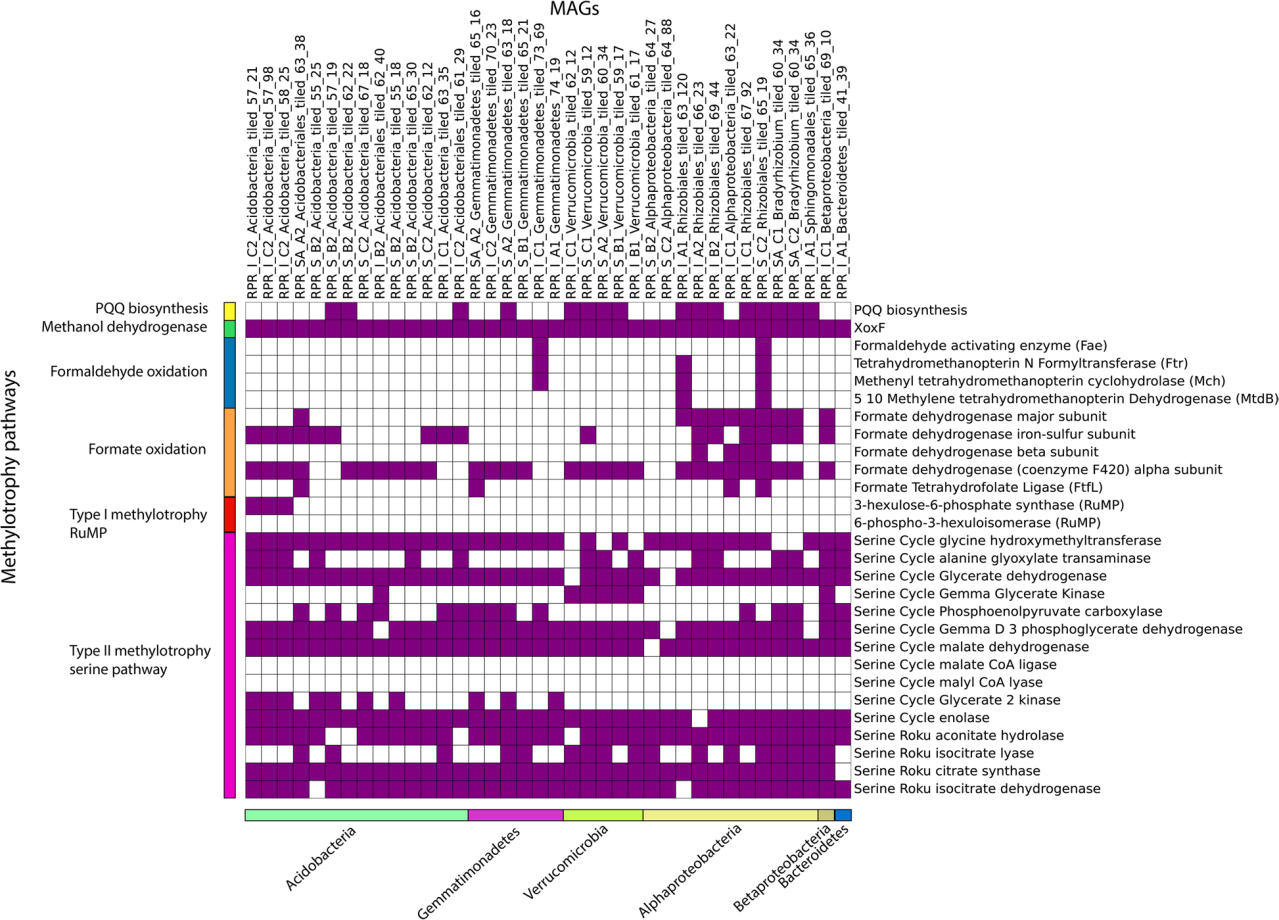
Methylotrophic genes of XoxF-containing MAGs. Columns represent methylotrophic MAGs and are grouped by their phyla at the bottom. Rows represent genes involved in methanol oxidation and are grouped into pathways on the left. The presence of a gene is indicated with a purple box and the absence is indicated with a white box

All 38 XoxF-containing genomes were analysed for methylotrophic genes (Fig. 8). All XoxF enzymes belong to the PQQ superfamily of dehydrogenases and use the PQQ redox cofactor in their active site. Sixteen genomes are capable of PQQ biosynthesis, while not all of the genomes can biosynthesise PQQ the cofactor may be obtained via PQQ-dependent TBDT [48, 49]. Following the oxidation of methanol (CH_3_OH) by PQQ MDH, the toxic formaldehyde (CH_2_O) must be converted to formate (HCOO^−^). Although the pathway for formaldehyde oxidation was only identified in three genomes, XoxF enzymes can oxidise formaldehyde [50]. Most of the genomes contain the genes necessary for formate oxidation and thus carbon dioxide production. The majority of the genomes also contain the entire serine pathway required to assimilate methanol. These results strongly support the capacity for lanthanide-dependent methylotrophy in the genomes.

## Discussion

Soils have been a major focus of metagenomic studies that address microbial community composition [16], community changes over depth and time [15, 26], biosynthetic potential [26], microbial carbon compound processing [51], response to climate change perturbation [52, 53], viral ecology [54] and many other aspects of biogeochemistry. However, microbial communities in weathered rocks—the precursors to soil—remain almost unstudied. Some exceptions include analysis of one metagenome of weathered shale [55] and two metagenomes in a weathered granodiorite [56]. In the current study, we analysed the metagenomes collected through a granite weathering profile. This approach enabled us to predict microbial utilisation of lanthanides in organic compound oxidation and to provide clues regarding links among microbial metabolism, phosphate mineral dissolution and lanthanide and phosphate bioavailability.

Microorganisms preferentially colonise mineral surfaces [57–59] and can assist in the breakdown of silicate [60–64] and non-silicate minerals [65–69], thereby enhancing rock weathering. Iron is an abundant redox active component of some minerals and microbial iron oxidation can promote mineral breakdown [56]. Microorganisms also assist weathering via production of acids [61]. However, lanthanides can precipitate as insoluble secondary lanthanide phosphates such as rhabdophane, as reported previously [6, 22, 40, 70] and as shown here, and these minerals are not solubilised via soil-associated acids [8]. Despite the low solubility of the lanthanide-containing minerals, we find that the lanthanide-bearing XoxF-type methanol dehydrogenases are highly abundant in weathered rock and soil. In fact, the XoxF-based system was the only methanol dehydrogenase type identified at our site, indicating that any methanol oxidation occurring was via lanthanide-based, instead of calcium-based, metabolism. Despite the significantly higher abundance and accessibility of calcium relative to lanthanides, XoxF may be favoured due its superior methanol oxidation activity [71]. The high abundance of these enzymes in the weathering profile, along with microscopic evidence indicating loss of lanthanide phosphate phases, suggests that the lanthanides in enzymes derive from co-existing lanthanide phosphate minerals. This raises questions about the mechanisms responsible for lanthanide acquisition and uptake from the environment.

Microbes can access elements such as Fe, which is highly insoluble under oxic conditions, using siderophores, which have high affinity for ferric iron and are capable of solubilising iron oxide minerals [67, 72, 73]. Long ago, the observation that soils were depleted in lanthanide phosphate minerals relative to underlying weathered rock motivated the suggestion that siderophores also may be required to dissolve lanthanide phosphate minerals [22]. Here we show that lanthanide phosphates are solubilised in moderately weathered rock (as well as soil), where genes for lanthanide-based enzymes are abundant. A recent preprint describes a lanthanophore shown to induce poorly soluble Nd_2_O_3_ dissolution and reports the first gene cluster implicated in this process [12]. The cluster is specific to *Methylobacterium extorquens* AM1 (Proteobacteria), and we find no related systems in bacteria with lanthanide-based enzymes in the weathered rock and soil studied here. Thus, we anticipate a variety of yet unknown siderophore-like molecules may perform the analogous function of complexing lanthanides to promote lanthanide phosphate mineral dissolution.

Our samples contained phosphate concentrations of < 100 ppm (Additional file 1: Supplementary Table 3), as is typical of low P soils and widespread in Australia [74]. This is, in part, due to phosphate precipitating as non-bioavailable insoluble secondary minerals such as the aforementioned rhabdophane and florence and iron phosphate (FePO_4_) (K_sp_1.3 × 10^−22^) [75], aluminium phosphate (AlPO_4_) (K_sp_ 1.3 × 10^−20^) [76], and calcium phosphate (Ca_3_(PO_4_)_2_) (K_sp_ 1.0 × 10^−25^) [77] in soils [78]. Lanthanide phosphate precipitation may be a widespread phenomenon, given that studies over many years have reported sequestration of P into lanthanide phosphate minerals during weathering of granitic rock [6, 22, 40]. It is possible that under phosphate limiting conditions, there are dual motivations for microbial solubilisation of lanthanide phosphates. Low phosphate may drive the production of secondary metabolites to dissolve rhabdophane and florencite thus making lanthanides bioavailable.

Metallophores are produced by large operonic sets of microbial biosynthetic gene clusters (BGCs). Genomes can contain numerous different BGCs, the products of which can be highly diverse, including metallophores, ionophores, antibiotics, antifungal compounds and signalling compounds [79]. Typically, it is challenging or impossible to discern the types of molecules that BGCs produce by bioinformatics analysis. However, recent analyses of gene clusters with known products showed that metallophore products of BGCs can be predicted by identifying NRPS/PKS gene clusters that contain TBDTs [41]. We resolved Acidobacteria genomes containing XoxF3 systems within putative metallophore clusters, which suggests these putative metallophores may be involved in the solubilisation and transport of lanthanides into cells. We also identified Acidobacteria genomes containing both XoxF3 and putative metallophore systems, although they were encoded in different genomic regions. Despite this, these putative metallophores may also assist in lanthanide solubilisation and uptake, given that the LCC of *Methylobacterium extorquens* AM1 is located distantly from the XoxF machinery [23]. The observation of genomes containing putative metallophore BGCs without XoxF systems in our site raises the possibility that some bacteria promote lanthanide phosphate mineral dissolution to access phosphorus, a byproduct of which is the release of lanthanides. Another possibility is that lanthanide-requiring microbes may be ‘cheaters’ [80] and able to modify metallophore compounds produced by other organisms, optimising them for lanthanide binding.

Given that lanthanides can have a toxic effect on bacteria [81, 82], it is equally possible that chelating molecules play a role in detoxification of lanthanides. However, bacteria may control lanthanide concentrations using metalloregulators of TBDTs and lanthanophores similar to the ‘lanthanide switch’ in which lanthanides control regulation of MxaF and XoxF in methylotrophs [83, 84].

To date, two transporters have been experimentally verified as required for lanthanide transport: the outer membrane siderophore-associated TBDT [19] and the periplasmic ABC-type transporter [85]. We identified two transport proteins not previously associated with XoxF systems, NRAMP and the mechanosensitive ion channel (MscS). The conserved co-occurrence of NRAMP with XoxF3 systems across Acidobacteria and Gemmatimonadetes indicate these genes may play a role in the transport of lanthanides. NRAMP proteins are involved in the transport of divalent metal cations such as Ca^2+^, Mn^2+^, Cd^2+^, Mg^2+^ and Fe^2+^ [86]. The five XoxF3 systems identified in Verrucomicrobia also contained a transporter channel not previously associated with XoxF systems. The MscS is a membrane channel that responds to cellular swelling when exposed to hypoosmotic solutions, by opening and allowing the cell to return to its resting volume [87]. These transporters have also evolved to include potential roles in Ca^2+^ regulation [88]. It would not be surprising if these channels play a role in lanthanide transport, given that Ca^2+^ and the lanthanides have similar atomic radii and the homology of calmodulin with lanmodulin [38] and MxaF with XoxF [14]. Given that these transporters are specific for metal cations rather than large organic molecules, and as lanthanide uptake involves a high affinity chelator [19], we suggest that these transporters uptake free lanthanides into the Verrucomicrobia, Acidobacteria and Gemmatimonadetes cells.

Topsoils can contain higher biosynthetic capacity than deeper soils [26]. This has been attributed to higher microbial interaction and competition in topsoils compared to deeper soils [89, 90]. However, this prior study did not include an analysis of weathered rock. In our study, microorganisms in the moderately weathered granite have the highest biosynthetic potential and this potential decreases with increased weathering. Further, we noted a higher incidence of NRPS/PKS BGCs in the moderately weathered rock relative to the heavily weathered rock and the soil. The pattern of metallophore-like BGCs decreasing with weathering zones is not supported by counts of metallophore with TBDTs. However, we note that TBDTs are exclusive to gram-negative bacteria and that other transporters may be involved [41]. We infer that metallophores may be required to access critical Fe, lanthanides and P in the weathered rock.

In the current study, we sampled a spheroidal weathered granite outcrop, the surface of which was colonised by lichens (Fig. 1), assemblages of organisms well known to promote early-stage rock weathering [91]. Some organic polymers secreted by members of lichen communities are likely stripped of their methylated groups by carbohydrate esterases, and thus a source of methanol [16]. Other sources of methanol include demethylation of pectin during growth of plant cells in surrounding vegetation and degradation of pectin and lignin [92]. Regardless of the source, methanol is ubiquitous in the atmosphere and terrestrial environments [93] and it is clear that methanol oxidation is an important microbial function during weathering of the granite studied here. Ultimately, oxidation of methanol in this zone leads to release of CO_2_ at the weathering front, and thus the production of carbonic acid. Therefore, lanthanide-using microbes, as well as heterotrophs whose growth is enabled by phosphate release, likely promote the dissolution of silicate minerals. The results reported here reveal how early rock colonisation may be linked to mineral weathering. A cascade of processes that result from phosphate and lanthanide element release will increase rock porosity and permeability, precursor steps for soil formation.

## Conclusions

Based on genome-resolved metagenomics conducted in mineralogical and geochemical context, we propose aspects of the complex biogeochemical processes of lanthanide acquisition, trafficking and utilisation in diverse bacteria across a weathered granite transect. Specifically, we report conserved gene clusters in bacteria from several phyla that would be suitable for experimental studies to describe new lanthanide-based systems. We find that lanthanide utilising microorganisms are prevalent within the region where insoluble lanthanide phosphate minerals dissolve and identify potential metallophore-like gene clusters that may be involved. Experimental characterisation of these systems may lead to routes for improving access to phosphorus in P-limited agricultural soils and for recovery of lanthanides from economical resources for technological applications.

## Methods

### Sample locations and sample collection

We collected 5 samples for geochemical analysis representing fresh and weathered rock and associated soil from an exposed I-type granite outcrop near Rocky Point Road (RPR) located near Ararat, Victoria, Australia. Samples were collected inwards from the outer, most weathered material, sampling along ‘zones’ where possible towards the freshest material. Samples ranged in texture from soil to highly (saprolite), moderately and lightly weathered material. The highly weathered material still retained their granitic texture. Six months later, we collected 9 samples for biological analysis around the same profile representing soil, highly weathered (saprolite) and moderately weathered granite. The geochemistry of these samples was also analysed. Samples were collected using a metal hand trowel sterilised using ethanol and flame. In the field, immediately after collecting the material, samples were homogenised, placed into sterile bags and flash frozen in a mixture of dry ice and ethanol and placed into an esky with dry ice for transport to the laboratory. Samples were delivered to the laboratory the same day and stored at − 80 °C before DNA extraction.

### Mineralogical sample preparation and analysis

Given that apatite is the most likely source for P required for REE/ P-bearing mineral precipitation, we focused on the surfaces of apatite crystals and relict apatite pits to locate secondary lanthanide phosphate minerals. Apatite crystals were identified in biotite grains with their c axes oriented parallel to the basal plane of biotite. As biotite contained euhedral apatite crystals up to 100 μm long, biotite grains were extracted from the weathered granite samples using tweezers under a binocular microscope and split along their cleavage plane using a scalpel to reveal interior basal planes. Biotite grains were used for apatite and secondary lanthanide phosphate characterisation using scanning electron microscopy (SEM) and energy-dispersive X-ray spectroscopy (EDX). Cleaved biotite grains were mounted on SEM stubs and glass slides using double-sided carbon adhesive, carbon-coated and secondary lanthanide phosphates were characterised using a FEI Teneo VolumeScope. Given their high average atomic number, REE/P-bearing minerals were located using back-scattered electron imaging, at an accelerating voltage of 10 kV. At least 50 biotite grains were examined per sample with anywhere from 0 to 30 secondary REE/P minerals analysed per sample. Mineral chemistry was determined using EDX analysis and was semi-quantitative and standardless with a predicted error rate of at least 10%. Mineral phase analysis was repeated and reproduced when possible to reduce experimental error. Sample density was determined by weighing samples, then coating them with parafilm and weighing samples in water.

### Whole-rock chemical analysis

Fist-sized rock samples were crushed using a rock crusher and then further milled into a powder using an agate ring mill. Whole-rock elemental analysis was performed using the Applied Technologies 7700 ICP-MS instrument with an expected error rate of ∼5%.

Fist-sized rock samples were crushed using a rock crusher and then further milled into a powder using an agate ring mill. Whole-rock elemental analysis was performed using the Applied Technologies 7700 ICP-MS instrument with an expected error rate of ∼5%. Samples were analysed at the School of Earth Sciences, the University of Melbourne on an Agilent 7700x. The instrument was tuned to give Cerium oxide levels of < 1%. There were 4 replicates of 100 scans per replicate measured for each isotope. Dwell times were 10 ms, except for Be, Cd, In, Sb, Ta, W, Tl and Bi, which were 30 ms. Long-sample washout times of 6 min with solutions of 0.5% Triton X-100, 0.025% HF in 5% HNO_3_ and 2% HNO_3_ and long-sample uptake times of 120 s were used. The USGS granite standard GSP-2 was analysed as unknown in each of the 4 runs. The average of these analyses agrees well with the long-term University of Melbourne average and, apart from Pr, multiple ID-TIMS and MIC-SSMS analyses by [94].

Using 100 mg of sample material, each sample was digested with HF-HNO_3_ mixtures in high-pressure bombs in an oven at 180 °C for 60 h. Solutions are then evaporated to dryness, then redissolved in HCl for 24 h in the oven. Next, samples were dried down and refluxed twice with concentrated HNO_3_, then dissolved in sealed vessels with 3N HNO_3_ overnight. Solutions were transferred to transparent polycarbonate tubes, diluted with water and centrifuged, then inspected for undissolved fluorides. If present, the supernatants are transferred to new tubes for those samples containing fluorides, and the fluorides are transferred to bombs and dissolved in HCl overnight in the oven. These solutions were then dried down, refluxed with nitric, then dissolved in 3N HNO_3_. These solutions were re-combined with their corresponding supernatant solutions and centrifuged to ensure no fluorides remained. An aliquot of the solution is further diluted with a 1.8% HNO_3_ solution containing an internal standard mixture to give a total dilution factor of 10,000. The amended analytical and drift correction procedures used are comprehensively described in [95]. The method uses a natural rock standard for calibration, internal drift correction using multi-internal standards (Li^6^, Sr^84^, Rh, Sm^147^, Re and U^235^), external drift monitors and aggressive washout procedures. Differences from [95] methods are (1) Tm, In and Bi were not used as internal standards as they are measured as analytes; (2) Two digestions of the USGS standard W-2 are used for instrument calibration. The preferred concentrations used for W-2 were mostly derived by analysing it against synthetic standards and a literature survey of isotope dilution analyses [96, 97]. Because only a single calibration standard is used, data can be easily normalised to other sets of preferred values for standards. Single element solutions were analysed after each run to measure isobaric interference levels to use in interference corrections.

### DNA extraction, sequencing and metagenomic assembly

DNA was extracted from 10 g of each sample using the PowerMax Soil DNA isolation kit (MoBio Laboratories). Metagenomic library preparation and DNA sequencing were performed at the Next Generation Sequencing Facility, Western Sydney University. Metagenomic libraries were prepared using the IDT Lotus PCR-free kit and sequencing was performed on a NovaSeq 6000 platform, producing 250 bp paired-end reads. From 9 samples, 18 metagenomes were produced and were processed individually. Raw reads were trimmed of adapters using bbduk (https://sourceforge.net/projects/bbmap/) with the following parameters: reference = Contaminants/adapters.fa k = 23 mink = 11 hdist = 1 tbo tpe ktrim = r ftm = 5. The reads were also screened for Phix and Illumina artefacts using bbduk and the following parameters: reference = resources/phix174_ill.ref.fa.gz,Contaminants/Illumina.articfacts.2013.12.fa.gz k = 31 hdist = 1. Finally, reads were quality trimmed using Sickle (https://github.com/najoshi/sickle) with the following parameters:: pe -q 20 -l 20. The samples were individually assembled using megahit with default parameters.

### Metagenome annotation

All samples were filtered to remove contigs smaller than 1000 bp using pullseq (https://github.com/bcthomas/pullseq). Open reading frames (ORFs) were predicted on all contigs using Prodigal v2.6.3 [98] with the following parameters: -m -p meta. Predicted ORFs were annotated using USEARCH [99] to search all ORFs against Uniprot [100], Uniref90 and KEGG [101]. 16S ribosomal rRNA genes were predicted using the 16SfromHMM.py script from the ctbBio python package using default parameters (https://github.com/christophertbrown/bioscripts). Transfer RNAs were predicted using tRNAscan-SE. The metagenomes and their annotations were then uploaded to ggkbase (https://ggkbase.berkeley.edu).

### Genome binning, filtering and dereplication

Metagenome assemblies were binned using differential coverage binners MaxBin2 [102], MetaBAT [103] and VAMB [104]. The highest quality bins from each metagenome were selected via DasTool [105]. Bin quality was manually assessed using the ggkbase platform based on contig coverage, GC values and the inventory of 51 bacterial single-copy genes. Bin completeness and contamination was also analysed using CheckM [106] lineage_wf using a threshold of > 70% completeness and < 10% contamination. Finally, bins were dereplicated at 98% nucleotide identity using dRep [107].

### Ribosomal protein S3 clustering and diversity analysis

All proteins predicted from the 18 metagenomes were analysed for rpS3 sequences using a custom hidden Markov model (HMM) from [16] with a threshold score of 40. Across all metagenomes, we identified a total of 3191 rpS3 sequences passed the assigned HMM threshold. Only rpS3 sequences with lengths of 180 to 450 amino acids were included resulting in 2181 rpS3 proteins. We then clustered the sequences at 99% ID using USEARCH to obtain clusters that approximately equate to species-level identification which we refer to as species groups. The following USEARCH command was used: -cluster_fast RPR_rpS3_filtered_seqs.faa -sort length -id 0.99 -maxrejects 0 -maxaccepts 0 -centroids RPR_rpS3_filtered_seqs_centroids.faa. This resulted in 1230 dereplicated rpS3 proteins, each approximately representing a species group. We then mapped the reads from each sample to the rpS3 bearing scaffold using BBMap for abundance quantifications. BBMap (http://sourceforge.net/projects/bbmap/) was then used to calculate the average coverage per base pair. The coverage was then normalised to percent abundances in each sample. rpS3 species groups were classified at the phylum level by constructing a phylogenetic tree containing our sequences and rpS3 references taken from the tree of life reference set [34]. Our 1230 rpS3 sequences were concatenated with the reference set and aligned using FAMSA. The resulting alignment was stripped of columns containing > 90% gaps using trimal and a phylogenetic tree was constructed from the alignment using FastTree. Sequences were then manually classified to the phylum level based on their position relative to reference sequences in the tree. A principle coordinate analysis (PCoA) was performed using the Bray–Curtis distance measure which was calculated using the R programming tool and the vegan package.

### Genome phylogenetic classification

To taxonomically classify the microorganisms represented by the 136 dereplicated bins, we used the combination of a concatenated ribosomal protein tree and a rpS3 protein tree. For the ribosomal protein tree, we searched each genome for 16 ribosomal proteins (RP16) using GOOSOS.py (https://github.com/jwestrob/GOOSOS). The following HMMs were used: Ribosomal_L2 (K02886), Ribosomal_L3 (K02906), Ribosomal_L4 (K02926), Ribosomal_L5 (K02931), Ribosomal_L6 (K02933), Ribosomal_L14 (K02874), Ribosomal_L15 (K02876), Ribosomal_L16 (K02878), Ribosomal_L18 (K02881), Ribosomal_L22 (K02890), Ribosomal_L24 (K02895), Ribosomal_S3 (K02982), Ribosomal_S8 (K02994), Ribosomal_S10 (K02946), Ribosomal_S17 (K02961) and Ribosomal_S19 (K02965). Ribosomal S10 model PF00338 was also used for identification of Chloroflexi. A total of 120 genomes containing at least 8 ribosomal proteins on a single contig were included. Our ribosomal protein sequences were then individually aligned using FAMSA and concatenated using the concatenate_and_align.py script from GOOSOS (https://github.com/jwestrob/GOOSOS/blob/master/Concatenate_And_Align.py). The resulting alignments were stripped of columns containing 90% gap positions using Trimal [108] with the parameter -gt 0.1. A phylogenetic tree was constructed using IQ-TREE and the following settings: iqtree -s RPR_RP16.mfaa -bb 1000 -nt AUTO -ntmax 48 -mset LG + FO + R. Genomes were then classified at the phylum level using GTDB-TK. If a genome was not included in the ribosomal protein tree, its taxonomy was determined via the rpS3 tree. For Acidobacteria genomes, class-level lineages were determined by building a ribosomal protein tree with 150 Acidobacteria reference genomes from [16] and manually classified based on their position relative to reference sequences in the tree.

### XoxF identification and classification

For methanol dehydrogenase (XoxF) identification and classification of clades, we constructed a phylogenetic tree to discriminate homologous, but functionally distinct proteins that cannot be identified by HMM search alone. XoxF sequences were identified in metagenomes using a custom HMM for PQQ-binding alcohol dehydrogenases taken from [16]. Across all metagenomes, we identified 927 XoxF sequences. Proteins greater than 300 amino acids in length were retained and dereplicated at 95% similarity using CD-HIT resulting in 411 XoxF sequences. These sequences were concatenated with a reference set [16, 55] and aligned using MAFFT using the following parameters: --localpair --maxiterate 1000 --reorder. The gaps were then removed from the alignments using trimal. A phylogenetic tree was constructed using FastTree, and sequences were manually classified based on their relationship with the XoxF reference set. The above method was repeated for the XoxF sequences derived from the dereplicated genome set resulting in 50 XoxF sequences. Regions of conservation in XoxF systems between bacterial genomes were identified using the progressive mauve genome algorithm in Geneious with default settings and the clinker gene cluster comparison tool [109].

### Biosynthetic gene cluster and metallophore prediction

To identify biosynthetic gene clusters (BGCs), antiSMASH 5.0 [110] was run on the metagenomes and the final set of dereplicated genomes using default parameters. BGCs retrieved from the metagenomes were dereplicated using CD-HIT at 95% and normalised to percent abundance in each sample. Only BGCs on contigs greater than 10 kb were included in the analysis from both the genomes and the metagenomes. The antiSMASH tool only classifies BGCs as siderophores when they contain IucA/IucC genes which are specific for aerobactin and aerobactin-like siderophores. To predict the occurrence of siderophores outside of aerobactin, we then ran two Pfams on the NRPS/PKS BGCs pfam_transporter20.hmm and all_sbp.hmm to identify the BGCs that contain the transporters: FecCD, Peripla_BP_2 and TonB_dep_Rec. Previous work has shown these transporters are predictive of siderophore activity [41].

### Supplementary Information

**Additional file 1: Supplementary Table 1.** All rpS3 groups. **Supplementary Table 2.** rpS3 gene coverage. **Supplementary Table 3.** All xoxF sequences identified in the metagenomes. **Supplementary Table 4.** Whole-rock elemental composition ICP-MS data. **Supplementary Table 5.** All BGCs (>10kbp) identified in the metagenomes. **Supplementary Table 6.** All BGCs (>10kbp) from the dereplicated genome set. **Supplementary Table 7.** EDX data for minerals in Fig. 6.

**Additional file 2: Supplementary Data 1.** Ribosomal protein S3 tree file.

**Additional file 3: Supplementary Figure 1.** Phylogenetic tree constructed with a concatenated alignment of 16 ribosomal proteins from 120 bacterial genomes resolved from this study. **Supplementary Figure 2.** Phylogenetic tree constructed with a concatenated alignment of 16 ribosomal proteins. **Supplementary Figure 3.** Gene cluster comparison of xoxF3 systems from Verrucomicrobia genomes. **Supplementary Figure 4.** Gene cluster comparison of xoxF3 systems from five Gemmatimonadetes genomes. **Supplementary Figure 5.** Predicted structure of the hypothetical proteins situated between CoxIII and CtaG in xoxF3 systems from Verrucomicrobia, Gemmatimonadetes and Acidobacteria genomes. **Supplementary Figure 6.** Phosphate regulons partially conserved across Acidobacteria and Gemmatimonadetes genomes recovered from the moderately weathered, highly weathered and soil regions. **Supplementary Figure 7.** ICP-MS data of all lanthanides and their concentration in the I-type RPR weathered granite profile relative to the freshest material (RPR8). **Supplementary Figure 8.** The total number and products of biosynthetic gene clusters (BGCs) predicted in scaffolds greater than 10kb from each metagenome (binned and unbinned sequences). **Supplementary Figure 9.** The percentage of biosynthetic gene cluster (BGC) products within taxonomic groups as predicted by AntiSMASH from the dereplicated genomes set.

**Additional file 4: Supplementary Data 2.** Multiple alignment of lanmodulin.

## Data Availability

The datasets generated and analysed during the current study are available in the DDBJ/END/GenBank repository under Project ID PRJNA1065000 (https://www.ncbi.nlm.nih.gov/bioproject/PRJNA1065000/) [111].

## Abbreviations

PQQ: Pyrroloquinoline quinone
TBDT: TonB-dependent transporter
MxaF: Calcium-dependent methanol dehydrogenase
XoxF: Lanthanide-dependent methanol dehydrogenase
MDH: Methanol dehydrogenase
BGC: Biosynthetic gene cluster
NRPS: Nonribosomal peptide synthetases
PKS: Polyketide synthases
LCC: Lanthanide chelation cluster
Cox: Cytochrome
RPR: Rocky point bushland reserve
SEM: Scanning electron microscopy
EDX: Energy-dispersive X-ray
rpS3: Ribosomal protein S3
DUF: Domain of unknown function
NRAMP: Natural resistance-associated macrophage protein
