# Supplementary material for: Weathered granites and soils harbour microbes with lanthanide-dependent methylotrophic enzymes
Source: BMC Biol. 2024 Feb 19;22:41. doi: 10.1186/s12915-024-01841-0 (PMC10875860; doi:10.1186/s12915-024-01841-0)
Supplement: Supplementary file 3 — Additional file 3: Supplementary Figure 1. Phylogenetic tree constructed with a concatenated alignment of 16 ribosomal proteins from 120 bacterial genomes resolved from this study. Supplementary Figure 2. Phylogenetic tree constructed with a concatenated alignment of 16 ribosomal proteins. Supplementary Figure 3. Gene cluster comparison of xoxF3 systems from Verrucomicrobia genomes. Supplementary Figure 4. Gene cluster comparison of xoxF3 systems from five Gemmatimonadetes genomes. Supplementary Figure 5. Predicted structure of the hypothetical proteins situated between CoxIII and CtaG in xoxF3 systems from Verrucomicrobia, Gemmatimonadetes and Acidobacteria genomes. Supplementary Figure 6. Phosphate regulons partially conserved across Acidobacteria and Gemmatimonadetes genomes recovered from the moderately weathered, highly weathered and soil regions. Supplementary Figure 7. ICP-MS data of all lanthanides and their concentration in the I-type RPR weathered granite profile relative to the freshest material (RPR8). Supplementary Figure 8. The total number and products of biosynthetic gene clusters (BGCs) predicted in scaffolds greater than 10kb from each metagenome (binned and unbinned sequences). Supplementary Figure 9. The percentage of biosynthetic gene cluster (BGC) products within taxonomic groups as predicted by AntiSMASH from the dereplicated genomes set. [file 12915_2024_1841_MOESM3_ESM.pdf]

Supplementary figures for “Weathered granites and soils harbour microbes with lanthanide-dependent methylotrophic enzymes”

Marcos Y. Voutsinos *et al.*

Corresponding author: Jillian F. Banfield, jbanfield@berkeley.edu

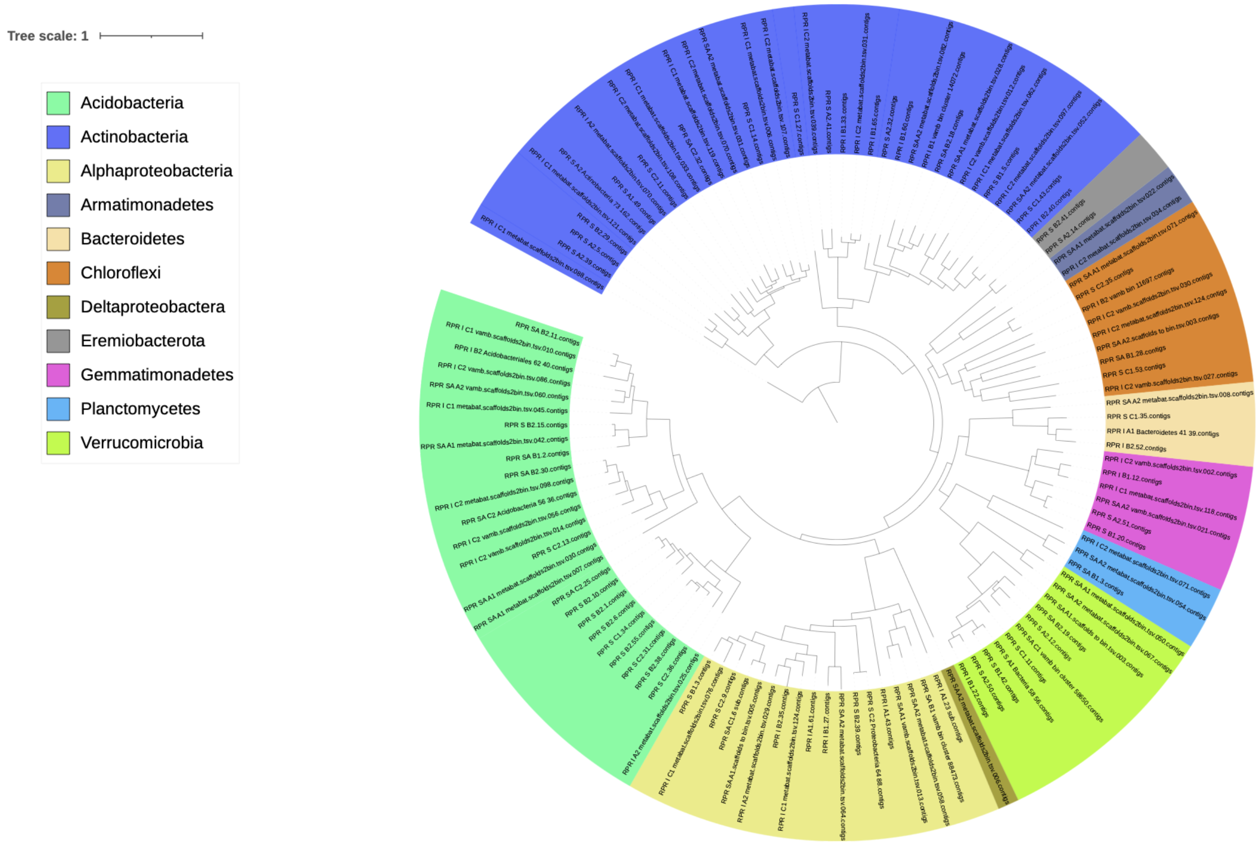

Supplementary Figure 1. Phylogenetic tree constructed with a concatenated alignment of 16 ribosomal proteins from 120 bacterial genomes resolved from this study. Genome names are coloured according to their phylum.

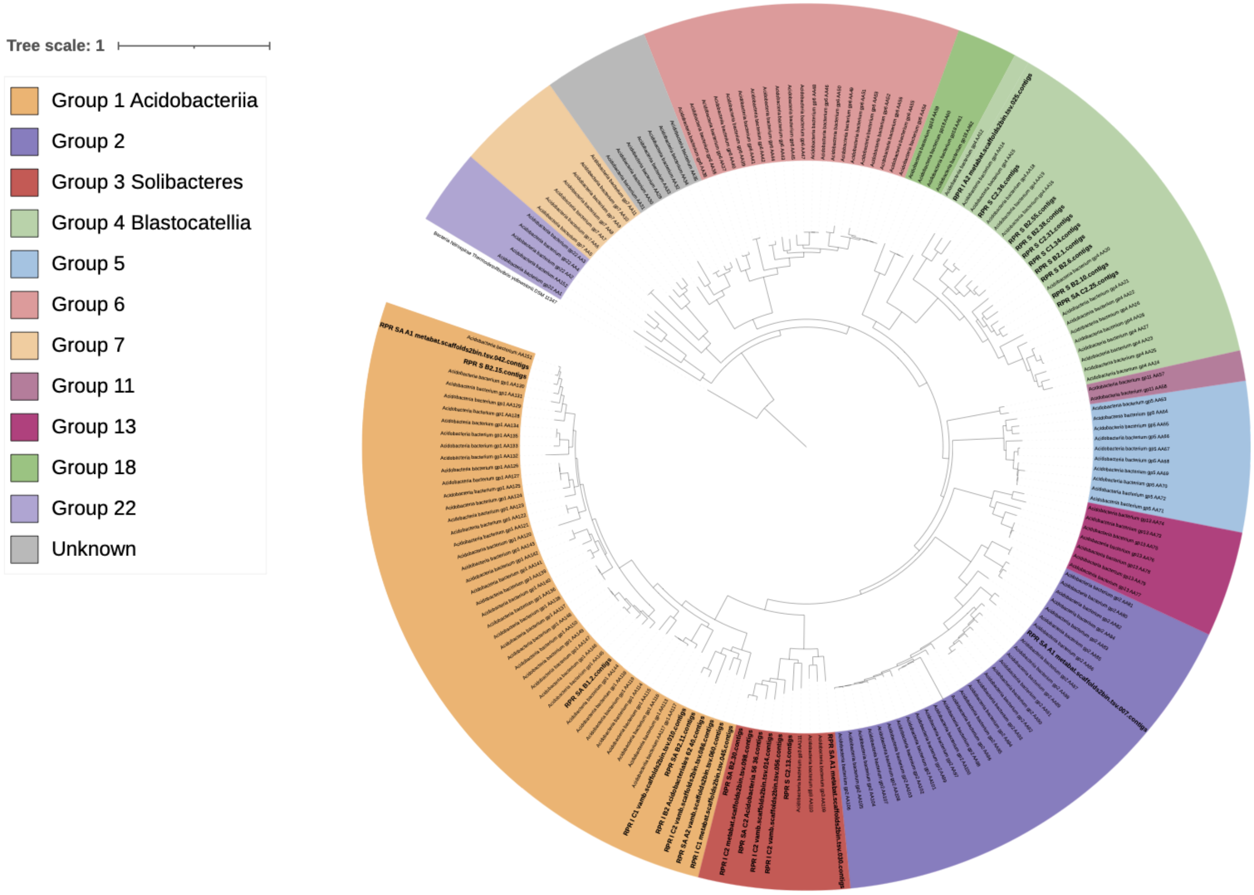

Supplementary Figure 2. Phylogenetic tree constructed with a concatenated alignment of 16 ribosomal proteins. The tree includes 28 acidobacteria bacterial genomes where 8 or more ribosomal proteins were identified from this study (bold) and 150 acidobacteria reference genomes from [16]. Nitrospira is present as an outgroup.

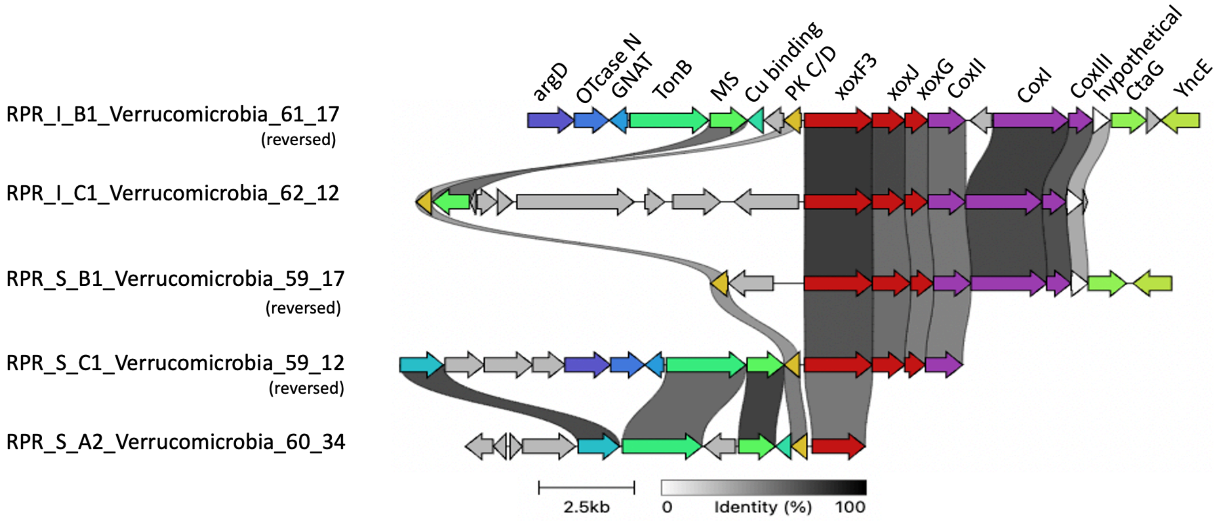

Supplementary Figure 3. Gene cluster comparison of xoxF3 systems from Verrucomicrobia genomes. Grey links show the percentage of identity between homologous proteins. Genes appearing once are grey and appearing twice or more are colour coded. Abbreviations in order of appearance: argD, acetylornithine aminotransferase; OTnase N, ornithine carbamoyltransferase; GNAT, N-acetyltransferase; TonB, TonB dependent receptor; MS, mechanosensitive ion channel; Cu binding, copper binding; PK C/D, polyketide cyclase dehydrase; xox, methanol dehydrogenase; Cox, cytochrome c oxidase; CtaG, cytochrome C oxidase assembly factor; YncE, PQQ-dependent catabolism-associated beta-propeller protein.

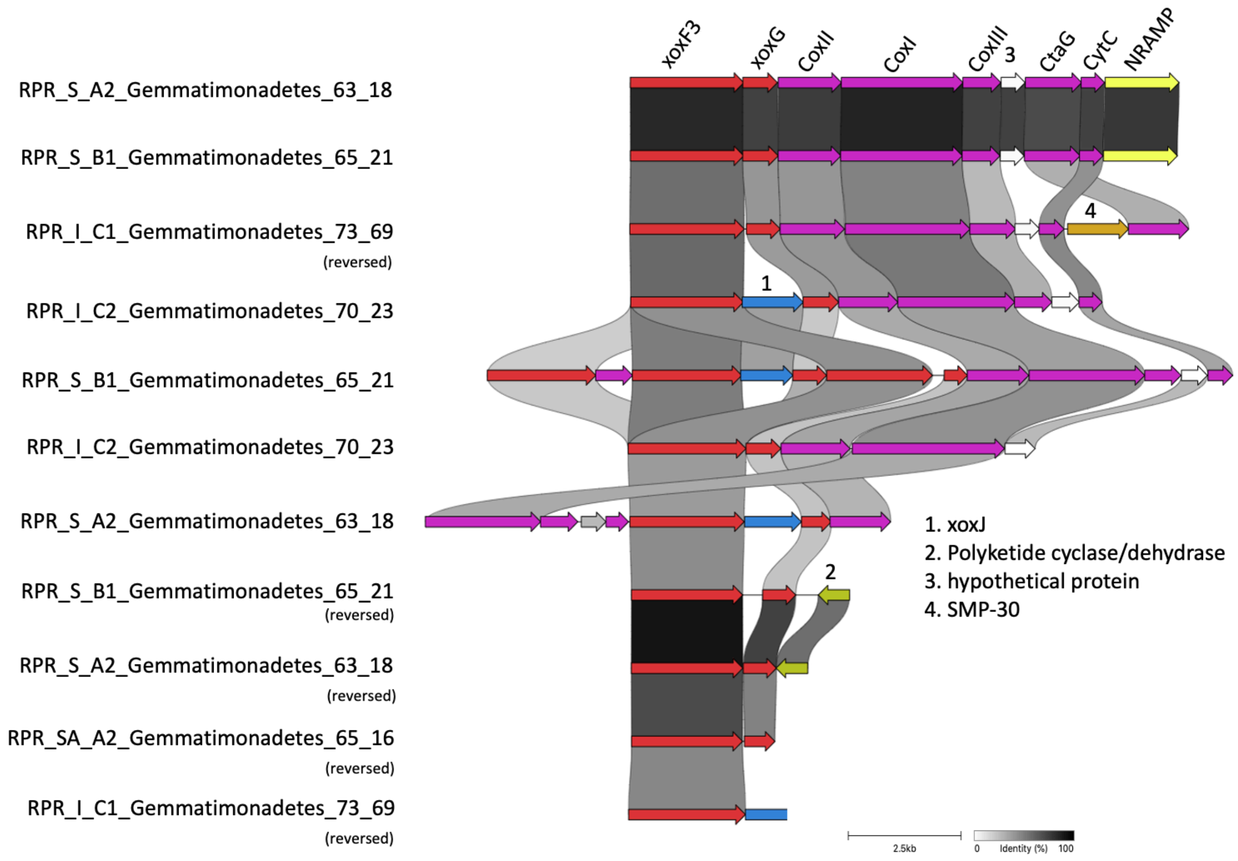

Supplementary Figure 4. Gene cluster comparison of xoxF3 systems from five Gemmatimonadetes genomes. Grey links show the percentage of identity between homologous proteins. Abbreviation in order of appearance: xox, methanol dehydrogenase; Cox, cytochrome c oxidase; CtaG, cytochrome c oxidase assembly factor; CytC, cytochrome c; SMP-30, senescence marker protein 30; NRAMP, natural resistance macrophage protein.

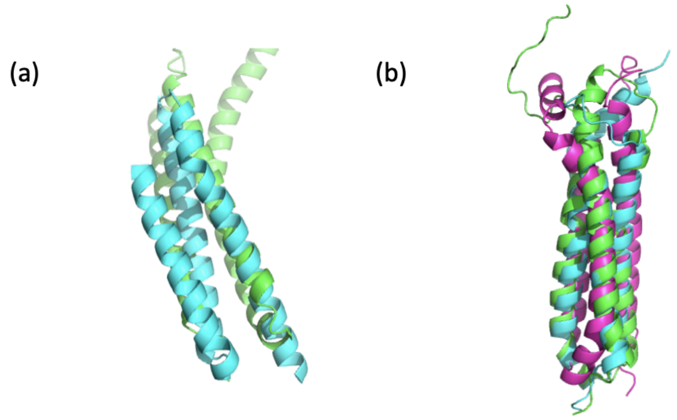

Supplementary Figure 5. Predicted structure of the hypothetical proteins situated between CoxIII and CtaG in xoxF3 systems from Verrucomicrobia, Gemmatimonadetes and Acidobacteria genomes. (a) Hypothetical protein (green) from RPR_S_B2_Acidobacteria_65_30 was superimposed in pyMOL with the best hit (cyan) PDB:2qyw (RMSD = 2.30) from PDBeFold. (b) Hypothetical proteins from RPR_I_B1_Verrucomicrobia_61_17, RPR_S_A2_Gemmatimonadetes_63_18 and RPR_S_B2_Acidobacteria_65_30 were aligned in pymol (RMSD = 1.170).

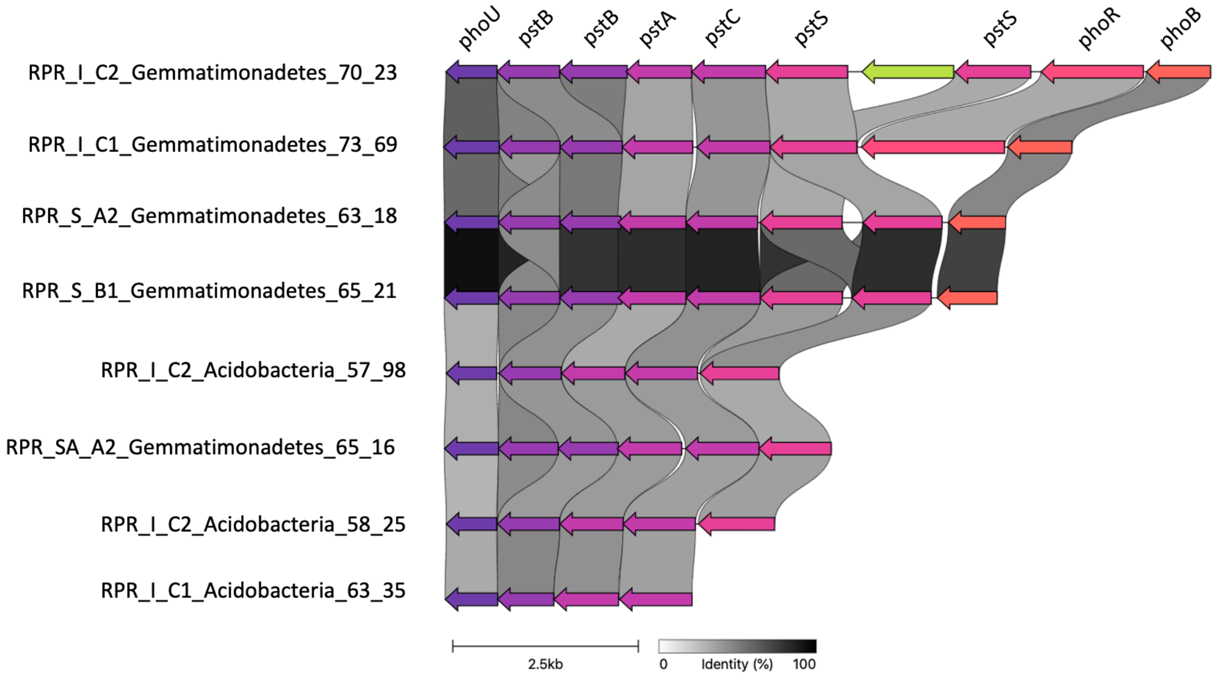

Supplementary Figure 6. Phosphate regulons partially conserved across Acidobacteria and Gemmatimonadetes genomes recovered from the moderately weathered, highly weathered and

soil regions. Grey links indicate the percentage of identity between homologous proteins from different genomes. Abbreviations in order of appearance: pho, phosphate uptake regulon; pst, phosphate specific transporter.

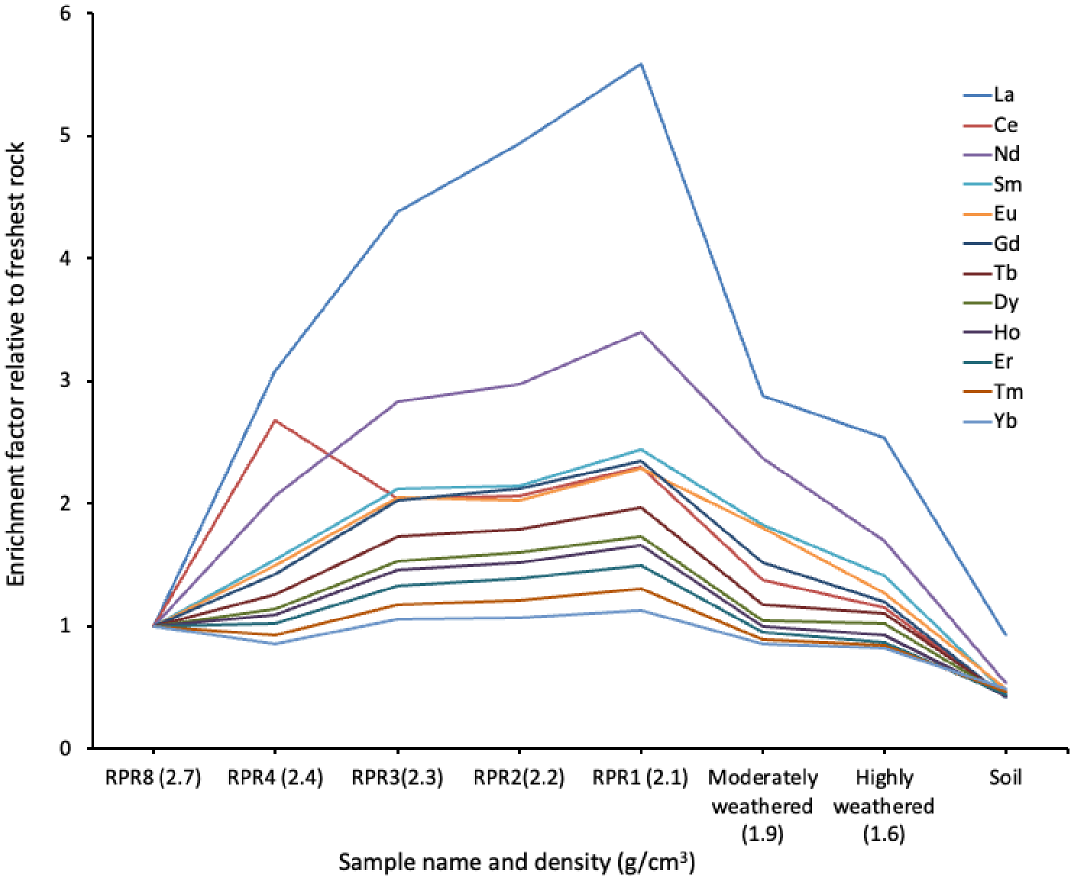

Supplementary Figure 7. ICP-MS data of all lanthanides and their concentration in the I-type RPR weathered granite profile relative to the freshest material (RPR8). Data are normalised to fresh rock to determine level of REE enrichment and expressed as “enrichment factor”.

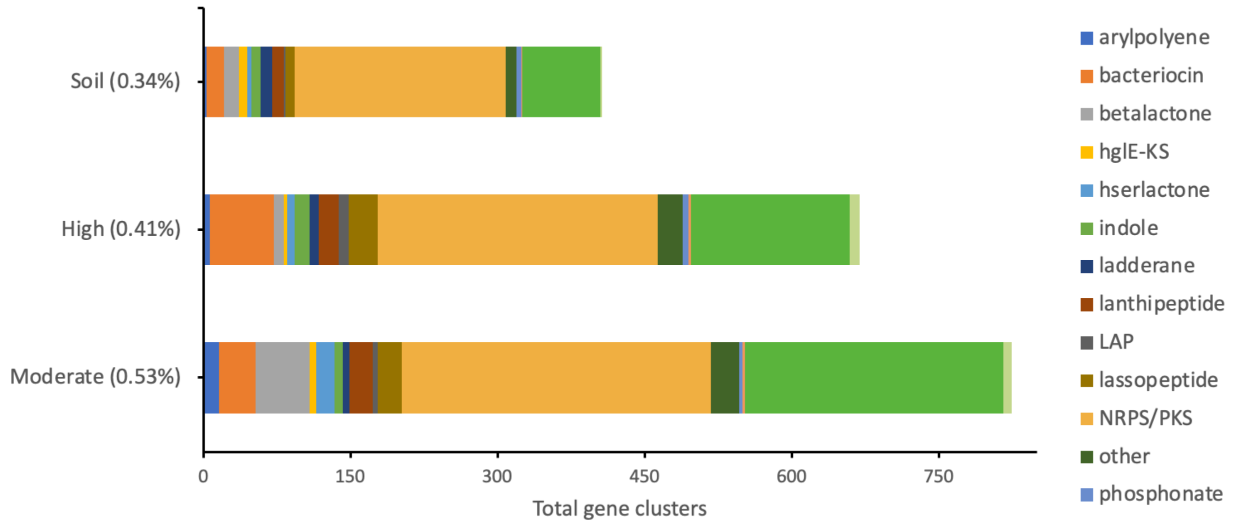

Supplementary Figure 8. The total number and products of biosynthetic gene clusters (BGCs) predicted in scaffolds greater than 10kb from each metagenome (binned and unbinned sequences) using antiSMASH 5.0. The number of BGCs from each weathered region were combined. BGCs (>10 kb) were normalised to percent abundance in each sample and are displayed beside sample names.

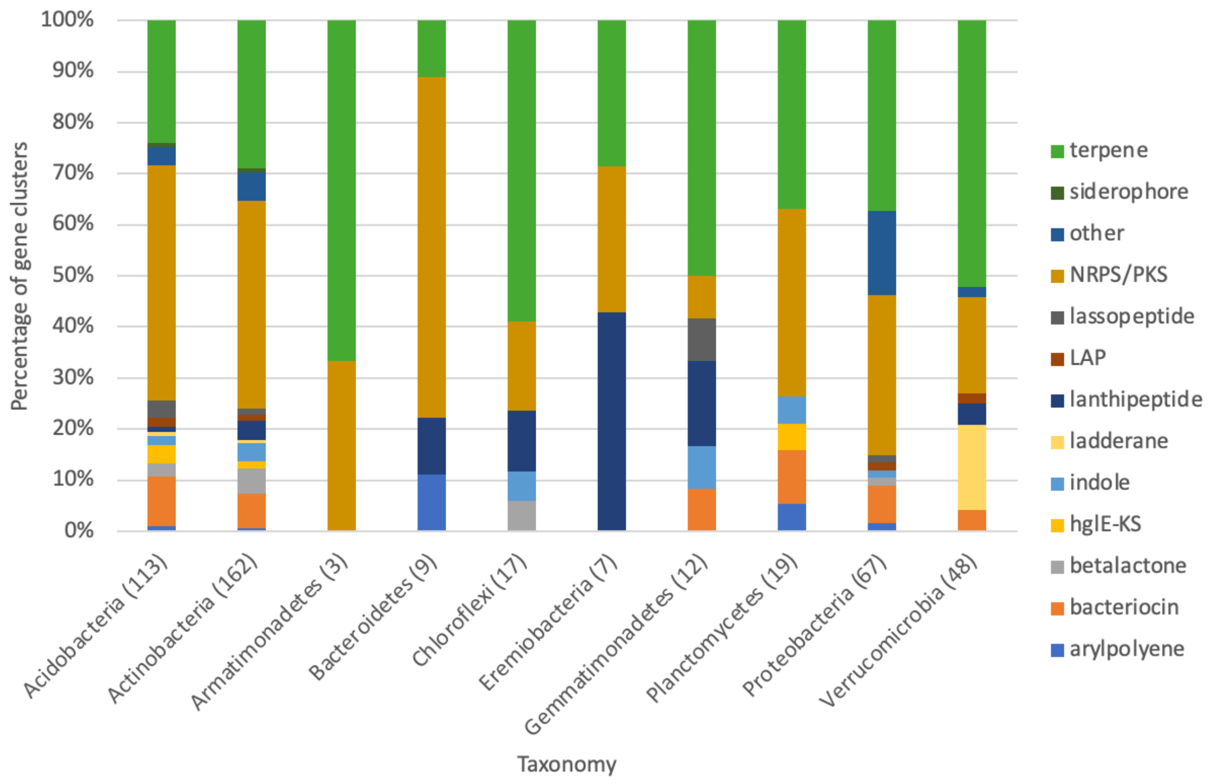

Supplementary Figure 9. The percentage of biosynthetic gene cluster (BGC) products within taxonomic groups as predicted by AntiSMASH from the dereplicated genomes set. Total number of BGCs in each taxonomy are presented in parentheses beside taxonomies. Only BGCs >10 kb are included.
